# Experimental Study on Surface Integrity of Solar Cell Silicon Wafers Sliced by Electrochemical Multi-Wire Saw

**DOI:** 10.3390/mi13091469

**Published:** 2022-09-04

**Authors:** Guanpei Bao, Chen Huang, Yajing Zhang, Zhen Yu, Wei Wang

**Affiliations:** 1 College of Mechanical Engineering, Anhui Science and Technology University, Huainan 233100, China; 2 College of Mechanical and Electrical Engineering, Nanjing University of Aeronautics and Astronautics, Nanjing 210016, China

**Keywords:** electrochemical multi-wire sawing (EMWS), silicon wafer, surface quality, surface damage layer

## Abstract

Electrochemical multi-wire sawing (EMWS) is a hybrid machining method based on a traditional multi-wire sawing (MWS) system. In this new method, a silicon ingot is connected to a positive electrode; the slicing wire is connected to a negative electrode. Material is removed by the interaction of mechanical grinding and an electrochemical reaction. In this paper, contrast experiments of EMWS and MWS were conducted based on industrialized equipment to verify the beneficial effects of the hybrid method. The experimental statistical results show that the composite processing method improved the processing qualification rate by 1.28%, and the Bow of silicon wafers was reduced by about 2.74 microns. Further testing on the surface of the silicon wafer after electrochemical action showed that obvious holes were present on the surface, and the surface hardness of the wafer decreased significantly. Therefore, the scratches on the surface of wafer sliced by EMWS were reduced; in addition, the thickness of the surface damage layer was reduced by about 9 microns. After standard texturing, the average reflectivity of the wafers sliced by EMWS was about 2–10% lower than that of the wafers sliced by MWS in the wavelength of 300–1100 nm. In this paper, the voltage parameter of the composite machining is set to 48 V; the amount of electrolyte added in each experiment is 2 L; and a good machining effect is obtained. In the future, the electric parameters and cutting fluid components will be further studied to improve the electrochemical effect.

## 1. Introduction

Silicon wafers are dominant substrate materials for the fabrication of microelectronics and solar cell components [1]. Owing to its many advantages, such as high cutting efficiency, small kerf width, and good surface equality, multi-wire sawing (MWS) gradually became the mainstream technology for wafer slicing for hard-brittle materials [2,3].

However, with the rapid development of intelligent equipment, the wafer industry is demanding better surface and subsurface quality, and higher processing efficiency. Meanwhile, thinner and larger diameter wafers will be a trend in the future [4,5]. Therefore, some new methods that combined conventional MWS have been applied to the manufacturing of silicon wafers.

The method of wire electrical discharge machining (WEDM) has been introduced to cut silicon in recent years [6,7]. On the basis of previous research results, a hybrid machining method that combined WEDM and fixed abrasive diamond wire sawing into one single process was proposed by Li. The research results showed that the hybrid machining method could improve the cutting efficiency, reduce the surface roughness and kerf width, and eliminate the recast layer and the surface heat-affected zone [8].

Ultrasonic vibration has great advantages in the processing of hard and brittle materials; thus, the wire sawing method that combined ultrasonic has been proposed [9]. Its superior performance has been confirmed by many researchers. Li et al. built an empirical model to predict the relationships between process parameters and surface roughness; experimental investigations were conducted. The experimental results showed that the surface roughness model could predict surface roughness with a relative error lower than 5%; moreover, optimal process parameters were obtained [10]. A theoretical model and verification experiments were conducted by Wang et al. for sawing force in an ultrasonic vibration-assisted diamond wire saw (UAWS) based on the theory of impact load. The measurement results indicated that the workpiece surface roughness of UAWS was 4.3%–29.7% lower than that of the conventional diamond wire saw [11].

A novel fixed and free abrasive combined wire sawing (FFACWS) technology was proposed by Gao et al.; an experimental study on the sawing characteristics was carried out. The results showed that within the range of the processing parameters, the obvious wire marks, parallel grooves, and ductile layers on the surface of the slices could be removed by FFACWS. The slices cut by FFACWS could be etched by mature acid etching technology [12,13].

**Figure 1:**
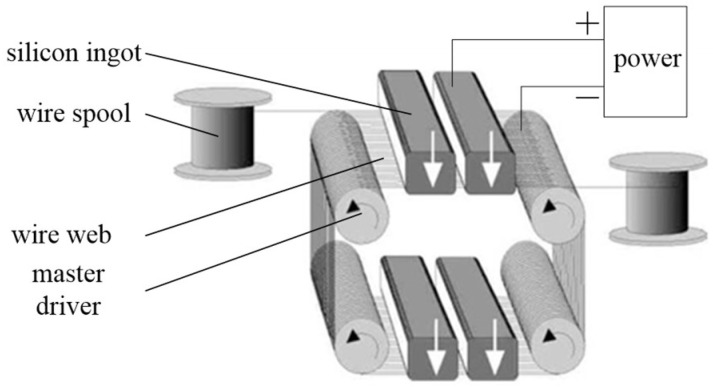
Schematic of EMWS.

Electrochemical MWS (EMWS) based on a MWS system was proposed by Wang [14]. The schematic of EMWS is shown in Figure 1. During the EWMS process, the metal slicing wire (cathode) and the silicon ingot (anode) are connected using a low-voltage continuous or pulsed direct current power supply.

The removal of material is achieved by the interaction of grinding and anodic oxidation. In the cutting zone, the silicon (connected to the anode) is oxidized under anodic potential first; then, the silicon substrate with oxide is chipped by abrasives.

The properties of the anodic layer caused by the electrochemical effect were analyzed. The results show that the anodic layer with a loose and porous structure was easily removed by the action of mechanical grinding [15]. Theoretically, this attribute is beneficial for minimizing cutting load and improving slicing efficiency. Unfortunately, the previous studies were all based on small-scale experimental equipment in the laboratory; thus, a certain gap exists with the industrial experimental equipment and system of the factory. Therefore, this paper conducts batch experiments based on industrialized equipment and performs statistical analysis of the experimental results. Then, the anodic oxidation experiment and analysis of a silicon wafer were conducted; the microstructure of the silicon wafer was analyzed; and the experimental results were verified.

## 2. Experimental Design

### 2.1. Experimental Setup

**Figure 2:**
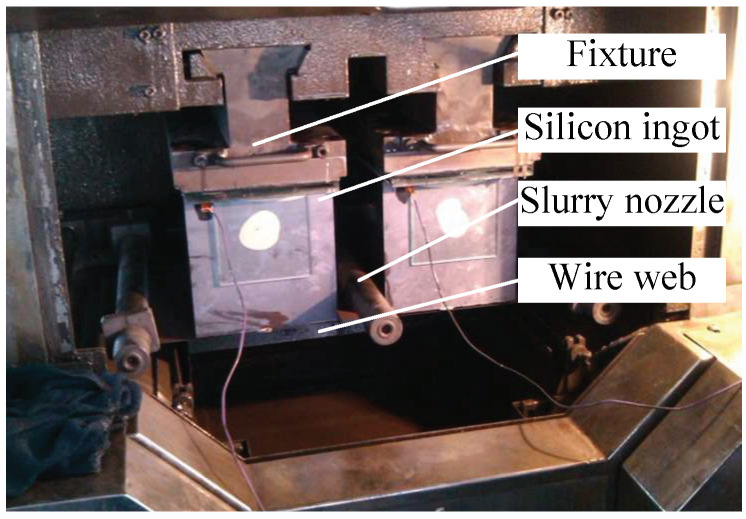
Photo of the experimental site.

The experiments are performed using a production-scale multiwire saw machine (NTC 442DM, Komatsu NTC Ltd., Nanto City, Japan), as shown in Figure 2. The pitch distance of the wire guide roller (WGR) is 315 µm, the wire diameter is 115 µm, and the size of SiC is 5–7 µm; accordingly, the thickness of the wafers is about 180 µm. The max wire velocity is 9 m/s, giving a total wire length of 180–200 km. The use of slurry that was composed of the polyethylene glycol (PEG) and SiC abrasives in a certain mass fraction is 200 kg for every slicing. Multi-crystalline silicon ingots with a similar property provided by GCL New Energy Holdings Limited are used for the EMWS and MWS experiments. To improve the accuracy of the experiments, batch slicing experiments are carried out. The EMWS experiments and the comparative experiments are conducted 10 times; in addition, the length of the silicon ingots is about 600 mm for every slicing. The silicon ingots, cutting wires, and slurry used in each experiment are chosen from the same manufacturer and the same batch. In these experiments, cutting wires were provided by Bekaert; slurry was provided by Henan Yicheng New Energy Co., Ltd., Kaifeng, China.

In comparison with the MWS system, the DC pulse power supply is the key difference of the EMWS system. In order to improve the effect of anodic oxidation and corrosion, a special electrolyte mainly composed of ethylene glycol and potassium chloride (1 mol/L) is added to the slurry during the process of EMWS. The amount of electrolyte added was 2 L for each experiment. The side of the silicon ingot is specially treated to reduce the contact resistance and realize electric energy transmission, as shown in Figure 2. The other parameters are the same. The main parameters of the experiments are summarized in Table 1. The electrical parameters for EMWS are summarized in Table 2.

### 2.2. Quality Measurement

**Figure 3:**
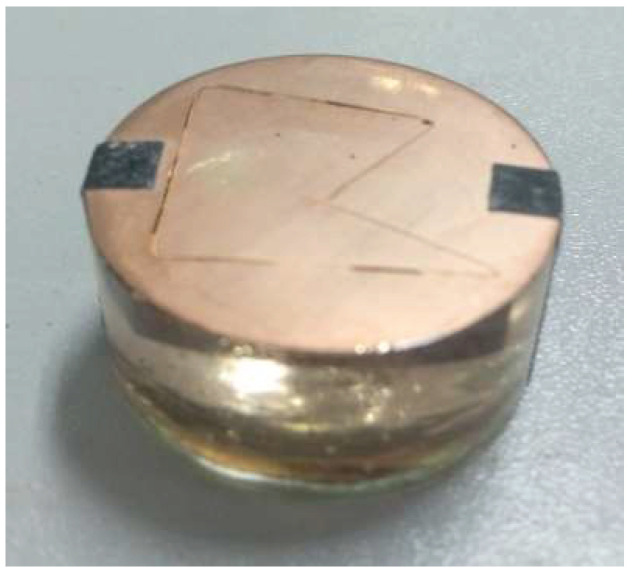
Picture of the test sample.

The surface quality of the silicon wafer affects the subsequent processing cost and the photoelectric conversion efficiency of the cell. Statistical analysis of the data and detection of the surface and sub-surface quality of silicon wafers can effectively evaluate the effect of the method. In these experiments, all the wafers were tested by the HENNECKE automatic silicon wafer sorter; the statistics are downloaded from it. The slicing surface and cross-sectional surface morphology were investigated by using a scanning electron microscope (SEM, Hitachi S-4800, Hitachi, Ltd., Tokyo, Japan). To detect the cross-section morphology, the silicon wafer is bonded to the support and then fixed in an epoxy mold. Then, the sample is polished with sandpaper, mechanically polished, and sprayed with gold. The prepared test sample is shown in Figure 3.

## 3. Results and Discussion

### 3.1. Statistics and Analysis of Machining Accuracy

**Figure 4:**
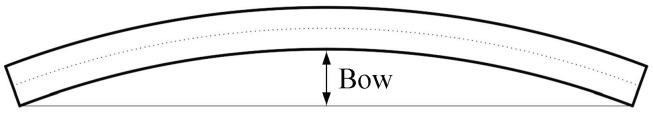
Schematic of Bow.

Machining accuracy is one of the important indexes to evaluate a process method. The main precision of silicon wafer includes thickness, total thickness variation (TTV), and bending of the bow (Bow). TTV represents the total thickness deviation of the silicon wafer, which refers to the difference between the maximum thickness and the minimum thickness of the silicon wafer. The wafer Bow is defined as the deflection from the neutral axis to the plane connecting the wafer edges, as shown in Figure 4 [16].

#### 3.1.1. Statistics and Analysis of Qualified Rate

From the analysis of material removal mechanism for EMWS, we speculate that this method can reduce the cutting load and improve the surface quality of wafers. Thus, contrast experiments are carried out to verify the good effects of EMWS on improving the bending of Bow, marks, and qualified rate.

Qualified rate is the most obvious indicator to evaluate a manufacturing method. Wafers are fabricated alternately by EMWS and MWS on one machine with the same process parameters. The dimensional accuracy requirements of qualified silicon wafers are as follows: 160 μm ≤ thickness ≤ 200 μm; TTV ≤ 30 μm; Bow ≤ 40 μm; saw marks ≤ 15 μm. The rate of broken wafers, saw marks, microcrack, disqualification of TTV, and qualified rate are counted and shown in Table 3. Under the same machining conditions, the qualified rate of EMWS is higher, and the rate of saw marks and microcrack are lower than that of MWS. The result shows that EMWS can avoid saw marks effectively. The main reason for the marks is the inadequate cutting capacity. Especially, marks often appear in the outlet position where few abrasives exist on the wire. The coupling actions of grinding and electrochemistry during EMWS can reduce the cutting load and increase the slicing ability; thus, the marks caused by poor slicing ability could be diminished.

#### 3.1.2. Statistics and Analysis of Bow

**Figure 5:**
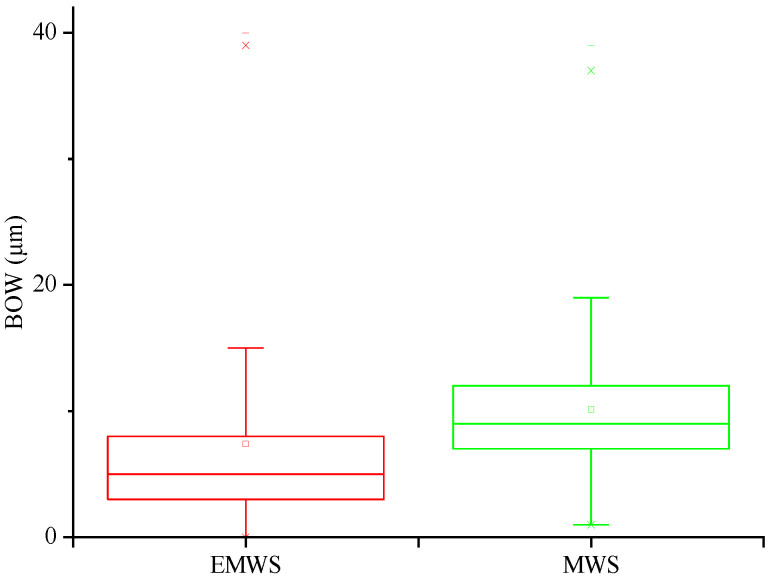
Distribution of Bow for EMWS and MWS.

Bow is one key indicators to evaluate the deformation and residual stress of wafers. On the one hand, the bending of the silicon wafer is caused by its own weight; in addition, the residual stress generated during processing is another important reason [17]. As the size of the wafers increases, the bow and warp worsen. Therefore, the bow should be strictly controlled. The statistics of Bow are shown in Figure 5. The results indicate that the average bow of MWS-sliced wafers is 10.12 μm, which is mainly distributed in the range from 5 μm to 14 μm; that of EMWS-sliced wafers is 7.38 μm, which is mainly distributed in the range from 0 μm to 9 μm. The bow of EMWS-sliced wafers is lower than that of the traditional MWS production. This is understandable in consideration of the material removal mechanism. The material removal of MWS completely depends on mechanical grinding, and residual stress could be produced because of work hardening. However, EMWS is a two-step process, i.e., oxidation and erosion by using an electrolyte and oxide removal by grinding; this can reduce the cutting load and improve the condition of work hardening.

### 3.2. Detection and Analysis of Microstructure

The statistics of machining accuracy can only reflect the changes of the statistical data; accordingly, intuitively explaining the reasons for the changes is difficult. To further explain the reasons for the data changes, the microstructure of the sliced wafers needs to be studied further.

#### 3.2.1. Analysis of Saw Marks

**Figure 6:**
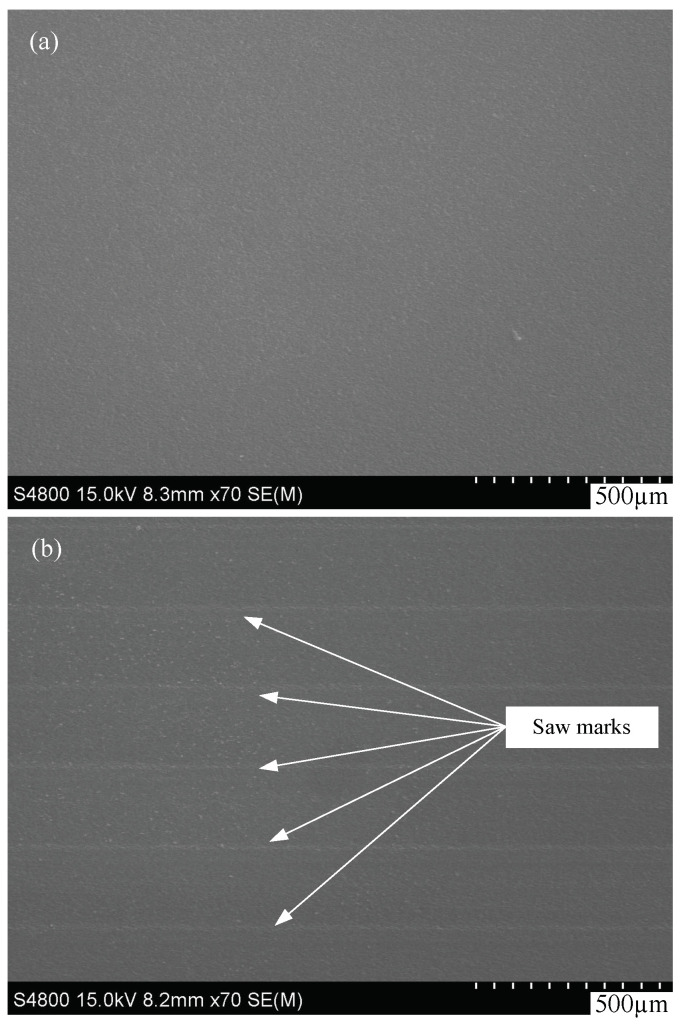
Surface topography of EMWS (**a**) and MWS (**b**).

In free abrasive wire slicing, the material is removed by the abrasives carried into the sawing channel by the wire. Unlike the ductile material removal method of a fixed abrasive wire saw, free abrasive cutting belongs to brittle removal; in addition, the surface of silicon wafer after cutting generally does not exhibit saw marks [18]. However, when the abrasives are not sharp enough or insufficient abrasives are carried to the cutting zone, saw marks will appear on the wafers. Nevertheless, even if these happen in EMWS, we can still obtain high-quality wafers without saw marks due to its special material removal mechanism. In the process of EMWS machining, the silicon in the cutting area is “changed” by the electrochemical action and could be sliced easily; this decreases the marks on the wafers. The typical surface topographies of wafers machined by EMWS and MWS are observed using a scanning electron microscope (SEM) and shown in Figure 6a,b. Compared with the saw marks that appear in Figure 6b, a smoother and more even surface is obtained by EMWS; this is shown in Figure 6a. The difference in surface topography implies different cutting mechanisms. The even surface generated by EMWS is attributed to “oxidation by using an electrolyte and oxide removal by grinding”.

#### 3.2.2. Analysis of Surface Damage Layer

In the machining process, the cutting wire drives the movement of abrasive particles; this results in transverse and median cracks on the subsurface of the silicon wafer, which will inevitably form a damage layer on the surface. Hence, the thickness of the cross-sectional damage layer on the silicon wafers sliced by MWS and EMWS is detected using a SEM to study the influence of different processing methods on the damage layer.

**Figure 7:**
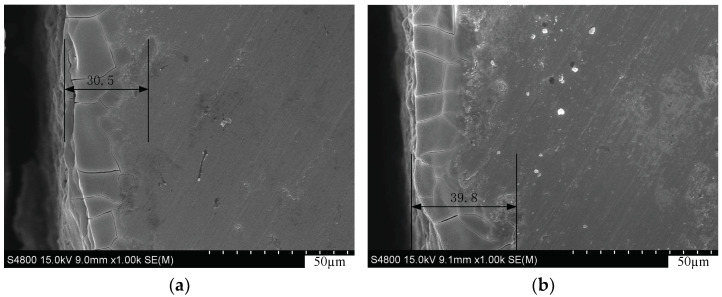
(**a**) Cross-section image of the wafer sliced by EMWS; (**b**) Cross-section image of the wafer sliced by MWS.

The cross sections of wafers sliced by MWS and EMWS are observed with a SEM (Hitachi S-4800, Hitachi, Ltd., Tokyo, Japan), as shown in Figure 7. The cross-sectional view in Figure 7b depicts that the cracks of the silicon wafer cut by MWS are dense, the depths of the cracks are inconsistent, and the deepest point is 39.8 µm. On the contrary, the cross-sectional view of the silicon wafer cut by EMWS shows that the cracks are sparse; and the depths are basically the same, about 30 µm. The reason is the electrochemical action softening the material in the cutting area and reducing the difficulty of cutting. Given that the damage layer will affect the service life and conversion efficiency of the cell, the damage layer should be removed in the production process. Owing to the electrochemical effect, the cutting load during EMWS is reduced, the thickness of the damage layer is reduced, and the wafer thinning amount in the cell manufacturing process is reduced; these factors are beneficial to reducing the production cost.

#### 3.2.3. Analysis of Anodic Oxide Layer

**Figure 8:**
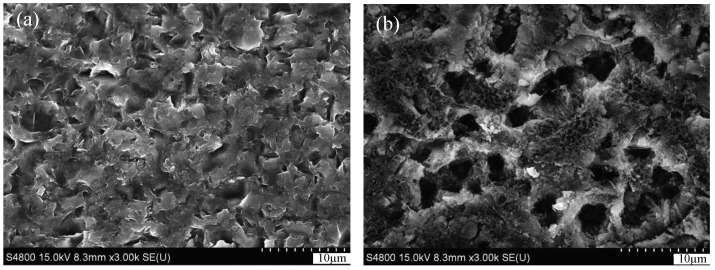
SEM images of the wafers with native oxide (**a**) and anodized oxide (**b**).

In order to study the effect of electrochemical action on the machining process, an additional experiment was conducted. A wafer manufactured by MWS was chosen and cut into two pieces. One wafer was exposed to air for the purpose of depositing native oxide on the surface; and the other wafer was dipped in the electrolyte that consists of ethylene glycol and potassium chloride at 48 V for 5 min to form anodic oxide. Then, the surface morphology of ultrasonically cleaned slices was observed by SEM. The surface hardness of the wafers was tested by using a TI 950 TriboIndenter. The SEM images (Figure 8a) reveal that the wafer with native oxide maintained its original characteristics. However, obvious holes appear on the surface of the silicon wafer after anodization; as shown in Figure 8b. Its hardness is only about 0.5 Gpa, which is much smaller than that of the natural oxide layer of 2.5 Gpa. The above test results prove that the hardness of the silicon wafer surface decreases after electrochemical action, and it is easier to remove.

### 3.3. Detection of Minority-Carrier Lifetime and Reflectivity

**Figure 9:**
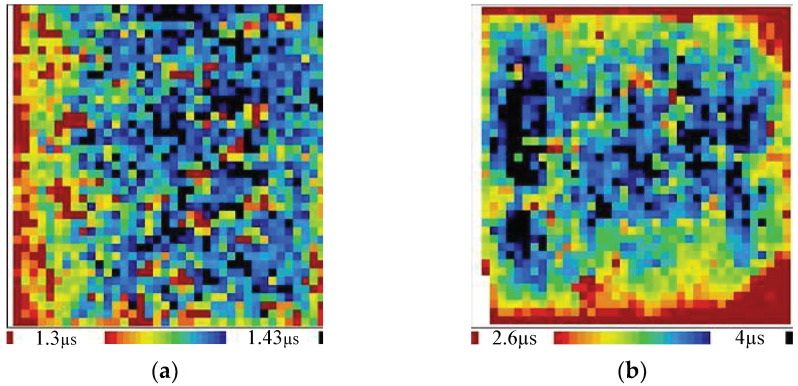
Minority carrier lifetime before (**a**) and after (**b**) texturization.

Minority carrier lifetime and reflectivity are quite important for the quality of solar cells. For solar cells, a longer minority carrier lifetime of the silicon wafer corresponds to a high photoelectric efficiency of the cell. Does the hybrid manufacturing method have any negative impacts on the minority carrier lifetime? Here, the minority carrier lifetime of wafers sliced by EMWS is measured using WT-2000 before and after texturing. The range of minority carrier lifetimes before texturing is from 1.33 μs to 1.45 μs, as shown in Figure 9a; after texturing, it is from 2.6 μs to 4.0 μs, as shown in Figure 9b. Both are in the normal range. Hence, this method has no adverse effect on the minority carrier lifetime.

**Figure 10:**
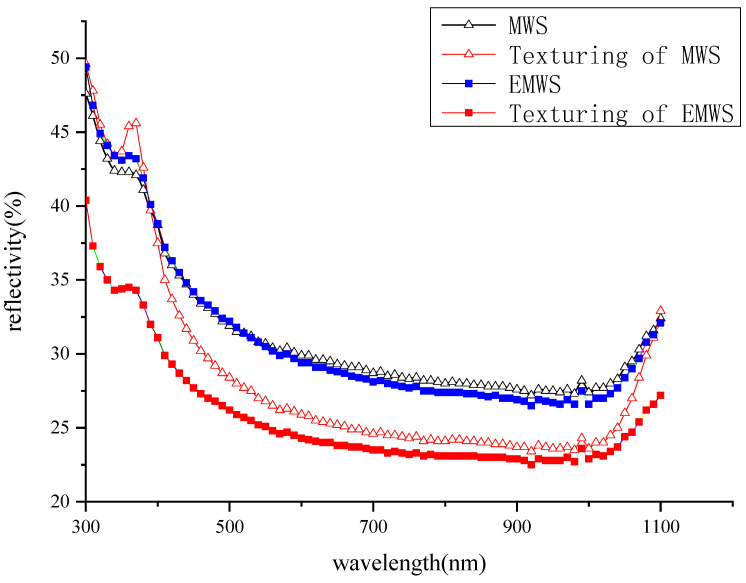
Reflectivity curves of the different silicon wafers.

The reflectivity of the silicon wafer after texturing is related to the conversion efficiency of the cell. In a silicon solar cell, lower optical reflectance significantly improves the minority carrier lifetime and photoelectric conversion efficiency by trapping more incident light [19]. Therefore, after standard texturing, the reflectivity of the wafers is measured. Figure 10 shows the reflectivity of the wafers based on different wire sawing technologies. Minimal difference in reflectivity exists between the EMWS-sliced wafer and the MWS-sliced wafer before texturization. However, the reflectivity of the EMWS-sliced wafer is about 2–10% lower than that of the MWS-sliced wafer after texturization. The reason is that the surface damage layer of the silicon wafer cut by EMWS is small. After the same texturing process is used, the surface damage layer is removed more thoroughly; thus, a better light-trapping structure is formed.

## 4. Conclusions

In this paper, a hybrid machining method that combines multi-wire saw and electrochemical was studied through comparative experiments based on industrial equipment. The surface morphology of the original silicon wafer and the anodized silicon wafer was detected to explain the processing mechanism. The hybrid machining method was studied through comparative experiments; moreover, analysis methods, such as statistics and detection, were used to evaluate its effectiveness. The following conclusions can be drawn based on the experimental results:

1. According to the statistical data of batch experiments, compared with the MWS method, the qualified rate of the EMWS method is increased by 1.28%; and the bow of the wafers is reduced by 2.74 µm, mainly distributed in the range of 0–9 µm.
2. The inspection and analysis of the silicon wafers indicate that the EMWS method can reduce the saw marks and the thickness of the surface damage layer. The surface of the anodic oxidation layer produced by electrochemical action is loose and porous; and its hardness is 0.5 Gpa, which is much smaller than the hardness of the natural oxidation layer of 2.5 Gpa. Therefore, the surface of the wafer that is cut by EMWS has fewer saw marks; in addition, the thickness of the surface damage layer is low.
3. The EMWS method has no negative effect on the minority carrier lifetime of silicon wafers. After the same texturing process is used, the surface reflectivity of the EMWS-sliced wafers is reduced by 2%–10% in the wavelength of 300–1100 nm compared with that of the MWS-sliced wafers.

Our findings show that the hybrid machining method can reduce the cutting load and improve the quality of silicon wafers under the corresponding experimental parameters (voltage of 48 V; electrolyte addition of 2 L for each experiment). This method is expected to be applied to the silicon wafer processing of solar cells and chips. In the future, the electric parameters will be further optimized and the components of the electrolyte will be studied. Further research will be conducted to study the influence of cutting fluid and electrical parameters on machining quality.

## Figures and Tables

**Table 1:** Experimental conditions for EMWS and MWS.

	EMWS	MWS
Material	P-type poly-Si	P-type poly-Si
Wafer size	156 × 156 mm	156 × 156 mm
Slurry	SiC + PEG	SiC + PEG
Wire tension	18 N	18 N
Wire	Φ115 μm	Φ115 μm
Wire speed	9 m/s	9 m/s
Feed rate	300 μm/min	300 μm/min

**Table 2:** Electrical parameters for EMWS.

	EMWS
Voltage	48 V
Peak current	25–30 A
Peak current density	1.1–1.5 mA/mm^2^
Period	500 μs
Pulse width	250 μs

**Table 3:** Comparison of the statistical data.

	Qualified (Wafers)	Qualified Rate (%)	Broken Wafer (%)	TTV (%)	Saw Marks (%)	Microcrack (%)
EMWS	19,198	93.23%	1.63%	1.08%	3.15%	0.19%
MWS	17,654	91.95%	1.59%	0.87%	4.50%	0.24%
